# Online application for the diagnosis of atherosclerosis by six genes

**DOI:** 10.1371/journal.pone.0301912

**Published:** 2024-04-10

**Authors:** Zunlan Zhao, Shouhang Chen, Hongzhao Wei, Weile Ma, Weili Shi, Yixin Si, Jun Wang, Liuyi Wang, Xiqing Li

**Affiliations:** 1 Department of General Medicine, Henan Provincial People’s Hospital, People’s Hospital of Zhengzhou University, Zhengzhou, Henan, China; 2 Department of Infectious Diseases, Children’s Hospital Affiliated to Zhengzhou University, Henan Children’s Hospital, Zhengzhou Children’s Hospital, Henan, China; 3 Department of Oncology, Henan Provincial People’s Hospital, People’s Hospital of Zhengzhou University, Zhengzhou, Henan, China; Southern Medical University, CHINA

## Abstract

**Background:**

Atherosclerosis (AS) is a primary contributor to cardiovascular disease, leading to significant global mortality rates. Developing effective diagnostic indicators and models for AS holds the potential to substantially reduce the fatalities and disabilities associated with cardiovascular disease. Blood sample analysis has emerged as a promising avenue for facilitating diagnosis and assessing disease prognosis. Nonetheless, it lacks an accurate model or tool for AS diagnosis. Hence, the principal objective of this study is to develop a convenient, simple, and accurate model for the early detection of AS.

**Methods:**

We downloaded the expression data of blood samples from GEO databases. By dividing the mean values of housekeeping genes (meanHGs) and applying the comBat function, we aimed to reduce the batch effect. After separating the datasets into training, evaluation, and testing sets, we applied differential expression analyses (DEA) between AS and control samples from the training dataset. Then, a gradient-boosting model was used to evaluate the importance of genes and identify the hub genes. Using different machine learning algorithms, we constructed a prediction model with the highest accuracy in the testing dataset. Finally, we make the machine learning models publicly accessible by shiny app construction.

**Results:**

Seven datasets (GSE9874, GSE12288, GSE20129, GSE23746, GSE27034, GSE90074, and GSE202625), including 403 samples with AS and 325 healthy subjects, were obtained by comprehensive searching and filtering by specific requirements. The batch effect was successfully removed by dividing the meanHGs and applying the comBat function. 331 genes were found to be related to atherosclerosis by the DEA analysis between AS and health samples. The top 6 genes with the highest importance values from the gradient boosting model were identified. Out of the seven machine learning algorithms tested, the random forest model exhibited the most impressive performance in the testing datasets, achieving an accuracy exceeding 0.8. While the batch effect reduction analysis in our study could have contributed to the increased accuracy values, our comparison results further highlight the superiority of our model over the genes provided in published studies. This underscores the effectiveness of our approach in delivering superior predictive performance. The machine-learning models were then uploaded to the Shiny app’s server, making it easy for users to distinguish AS samples from normal samples.

**Conclusions:**

A prognostic Shiny application, built upon six potential atherosclerosis-associated genes, has been developed, offering an accurate diagnosis of atherosclerosis.

## 1. Introduction

Atherosclerosis (AS) is the most common cause of peripheral vascular disease, coronary heart disease, and cerebral infarction [1]. In many countries, it serves as the leading risk factor for half of all deaths [2]. This disease is marked by the development and accumulation of atherosclerotic plaque in the vessel wall of arteries [3], thereby impairing arterial function. Endothelial-cell damage serves as the main trigger for the development of atherosclerotic plaque, and an inflammatory-fibroproliferative response is caused by various forms of damage to the endothelium [4]. Atherosclerotic plaque progression occurs at an accelerated pace in individuals who possess risk factors such as high blood pressure, smoking, diabetes, obesity, and genetic predisposition [5]. Various diagnostic techniques, including invasive methods like selective coronary angiography and non-invasive approaches such as blood biomarkers, enable the evaluation of cardiovascular disease risk and treatment objectives [6]. Of these methods, blood biomarkers are often preferred due to their non-invasive nature, convenience, and cost-effectiveness. However, it is worth noting that there is currently no readily available and highly accurate model for the diagnosis of atherosclerosis.

Due to the progress in omics technology and the increased accessibility of clinical blood samples, several investigations have concentrated on analyzing the blood transcriptome of individuals with AS [7]. The examination of blood cell transcriptomes between AS and control subjects holds the potential to uncover valuable diagnostic biomarkers. Concurrently, biomedical literature has witnessed a significant increase in studies integrating machine learning techniques [8]. These studies have demonstrated the superior performance of machine learning over traditional statistical approaches in terms of precision and predictive capacity [9]. For instance, a study employed machine learning to predict the prognosis of cancer survival by analyzing gene expressions [10]. While specific biomarkers were indeed identified [11], the precision of the machine learning models on AS was notably absent.

A noteworthy problem with the machine learning models on AS diagnosis is the small sample sizes. To address this point, integrating multiple datasets into one dataset will significantly improve the sample size. However, the non-biological variables among datasets, such as platforms and techniques, usually can be referred to as the “batch effect”, which is an obstacle to integrating datasets. Some bioinformatics strategies, such as the comBat function, have been developed for microarray data batch correction [12]. The comBat function employs an empirical Bayes approach to correct data for batch effects, demonstrating robustness across datasets [13]. However, this method has been rarely applied to analyze AS blood samples.

In this study, DEA and the gradient boosting model were used to screen possible genes for AS diagnosis after correcting the batch effect and integrating multiple datasets. The top 6 genes from the gradient boosting model were identified. Random forest performed best in the evaluation and testing datasets among seven machine learning algorithms. Comparison results also showed that our model performed better than the genes provided in the published studies. The final machine learning models were then uploaded to Shiny’s server, which allows users to easily distinguish AS samples from control samples.

## 2. Methods

### 2.1. Collection of mRNA expression data

Genomic expression profiles for atherosclerosis and matched controls were sourced from the GEO database. The sample count was limited to a range of 10 to 1000. The AS microarray dataset was selected according to the following criteria: (1) atherosclerosis in patients with blood samples; (2) at least five samples per group. (3) datasets with more than 10,000 gene expression profiles available. Using the selection above criteria, seven datasets were ultimately incorporated into the study: GSE9874, GSE12288, GSE20129, GSE23746, GSE27034, GSE90074, and GSE202625. The fundamentals of these microarray datasets are listed in **Table 1**. The expression data in these datasets were obtained on 10 July 2023. There was no information available that could identify individual participants.

**Table 1:** The information of datasets in this study.

Dataset	Platforms	Sample number	AS samples number	Normal samples number	Publication
GSE9874	GPL96	60	30	30	[31]
GSE12288	GPL96	222	110	112	[32]
GSE20129	GPL6104	119	48	71	[33]
GSE23746	GPL2700	95	76	19	[34]
GSE27034	GPL570	37	19	18	[35]
GSE90074	GPL6480	143	93	50	[36]
GSE202625	GPL23934	52	27	25	[37]

### 2.2. Data processing

Since seven different datasets were included in our study, batch effects among datasets are inevitable. Several methods have been employed or devised to detect and eliminate batch effects from genomic datasets. Among these techniques, ComBat uses an empirical Bayesian approach to correct batch effects and has demonstrated superior performance compared to other methods [14]. In this study, we introduced the housekeeping genes alongside the ComBat function to remove the batch effect. Housekeeping genes are essential for cellular activities, irrespective of their unique function within the tissue or organism. As such, they are expected to be consistently expressed across all cells under different conditions, regardless of tissue variety, developmental phase, or cell cycle status. The process of removing the batch effect in our study was as follows: (1) The normalized gene expression data was downloaded by the “getGEO” function from the ‘GEOquery’ package [15]. If the normalized gene expression data is not available, the non-normalized expression data, which is often contained in the supplementary file on the corresponding website of the NCBI GEO database, can also be used. We selected the probe with the highest expression value for multiple probes corresponding to the same gene. (2) For some normalized expression data, the min value in the expression matrix could be negative. A value was added to ensure that the minimum value in the expression matrix is positive. (3) The housekeeping genes list was downloaded from the previously published article [16], which contains 2158 housekeeping genes. Then, we calculated each sample’s mean expression value of these 2158 housekeeping genes (meanHGs). Higher meanHGs value in a sample indicates an over-expression of the whole genome than other samples, which is regarded as the primary source of the batch effect. To reduce this over-expression of the batch effect, all gene expression values in a sample were divided by the corresponding meanHGs of this sample. (4) The expression data, normalized by dividing with meanHGs, were corrected using the comBat function. Finally, the expression datasets were selected for further analysis after batch correction. Principal component analysis (PCA) and t-distributed stochastic neighbor embedding (tSNE) were used to visualize data structure and the batch between datasets.

### 2.3. Data division and differential expression analyses

This analysis scrutinized seven distinct datasets: GSE9874, GSE12288, GSE20129, GSE23746, GSE27034, GSE90074, and GSE202625. Two datasets, namely GSE202625 and GSE12288, were chosen to serve as independent testing grounds. This strategic selection was aimed at ensuring a robust validation of the findings. The samples from the five datasets not designated for testing were divided. Specifically, a random selection process was employed to allocate precisely half of the samples to form the training dataset. This dataset is crucial for developing and refining the analytical models. The remaining 50% of the samples were assigned to the evaluation dataset. This division was crafted to comprehensively assess the model’s performance, allowing an in-depth understanding of its predictive capabilities and overall accuracy. This approach underscores the meticulous planning and thoughtful consideration invested in the study’s design, aiming to maximize the reliability and validity of the outcomes.

We performed a T-test analysis for the differential expression analyses (DEA) between AS and control samples. The DEA analysis was conducted using the training dataset. Genes with adjusted p-value < 0.05 (Bonferroni correction) were selected as differentially expressed genes (DEGs) between AS and control samples. It is worth noting that we removed the housekeeping genes from the gene expression matrix before the DEA analysis.

### 2.4. Functional enrichment analysis

Gene Ontology (GO) terms, including "GO_Biological_Process_2021", were analyzed using the enrichR package for the DEGs [17]. WikiPathways is a collaborative platform that allows the community to contribute and maintain biological pathways. The "WikiPathways_2019_Human" dataset within WikiPathways contains pathway information related to the human genome. The Molecular Signatures Database (MSigDB) is a collection of gene sets representing well-defined biological states, cellular processes, and pathways. "MSigDB_Hallmark_2020" contains a set of gene signatures representing hallmark biological processes. Enriched items with adjusted p-values of less than 0.05 were considered statistically significant.

### 2.5. Screening and verification of diagnostic markers

To identify candidate hub genes, we calculated each feature’s relative importance (ReImp) using the gradient boosting model on the training dataset. The ReImp indicates the impact and importance of each predictor variable during the model training process. We then plotted the top 10 genes by their overall feature importance from high to low.

### 2.6. Evaluation of seven algorithms

Seven algorithms, including Generalized linear Model (GM), K nearest Neighbors (KN), Linear discriminant Analysis (LA), Naive Bayes (NB), Quadratic discriminant Analysis (QA), Random Forest (RF), Support vector Machine (SM), were selected for the model construction. The optimal parameters for each algorithm were determined through 5-fold cross-validation. The training control parameters set for the machine learning model include method = ’cv’, indicating the use of cross-validation; number = 5, specifying the number of cross-validation folds; classProbs = TRUE, to have class probabilities; and search = ’random’, for conducting a random search in parameter tuning. Receiver operating characteristic curve (ROC) curves were generated to assess the diagnostic potential of each candidate gene. Furthermore, evaluation metrics such as Area Under the Curve (AUC), accuracy, sensitivity, specificity, precision, and F1-score were computed for both the evaluation and testing datasets.

### 2.7. Comparison with the current biomarkers

A common approach for diagnosing AS involves combining gene expression data with machine learning models. Thus, we compared our results described above with those of a classifier based on gene expression. Accuracy and AUC values were used to compare studies. A total of 15 studies were searched and selected. Bin et al. used protein interaction (PPI) networks to identify six genes related to the progression of atherosclerosis and regulate immune cells [18]. Cheng et al. identified eight pivotal genes by analyzing hub differentially expressed genes [19]. Chunjiang et al. selected three hub genes for diagnosing carotid plaque progression [20]. Di et al. used bioinformatics analysis to identify seven genes essential to late-stage carotid atherosclerosis markers [21]. Feng et al. selected ten genes to predict normal and atherosclerosis samples [11]. Jing et al. selected four pivotal genes that could affect the progress of AS based on PPI [22]. Julong et al. screened ten hub genes that may play a crucial role in the progression of histologically unstable carotid plaques [23]. Rong et al. identified six core genes linked to atherosclerotic plaque progression in the protein-protein interaction (PPI) network [24]. Shihuan et al. developed a three-gene model to evaluate the prognosis of AS [25]. Yajuan et al. identified three critical genes diagnostic biomarkers of AS [26]. Yongjiang et al. identified three correlated biomarkers for the diagnosis of atherosclerosis [6]. Yue et al. screened eight hub genes that may predict plaque progression [27]. Yujia et al. identified seven characteristic genes that enabled the prediction of the progression of atherosclerotic plaques [28]. Zhen et al. identified three genes as diagnostic markers for AS [29]. Zhipeng et al. established that CD52 and IL1RN may play a vital role in the occurrence and development of atherosclerosis [30].

The genes in the articles above were obtained. We used the same training and testing dataset to validate the performance of these genes. We selected the same algorithm (random forest) to construct the model since random forest got the best performance in the testing dataset. As with the previous steps described in Section 2.6 "Evaluation of seven algorithms", the optimal parameters for each algorithm were selected using 5-fold cross-validation with random search. Moreover, we rebuilt our model during this process to ensure a fair comparison. AUC and Accuracy values in the testing dataset were calculated to evaluate each candidate gene combination’s diagnostic value.

### 2.8. The construction of Shiny apps based on machine learning models

R users can easily create interactive web apps using Shiny’s robust framework. This technology makes the creation of dynamic visualizations, current dashboards, data exploration tools, model presentations, sophisticated workflow applications, and more possible. The user interface (UI) and server are the two main components of Shiny software. The server determines the app’s behavior and functionality, but the user interface defines its appearance and display. Creating responsive apps that update and change in response to user input is easy using Shiny. We used seven models that had been made in our study. Based on six genes, the Shiny application we built seeks to assist users in predicting AS samples against normal ones.

## 3.Results

### 3.1. Flowchart and correction of the batch effect

**Fig 1:**
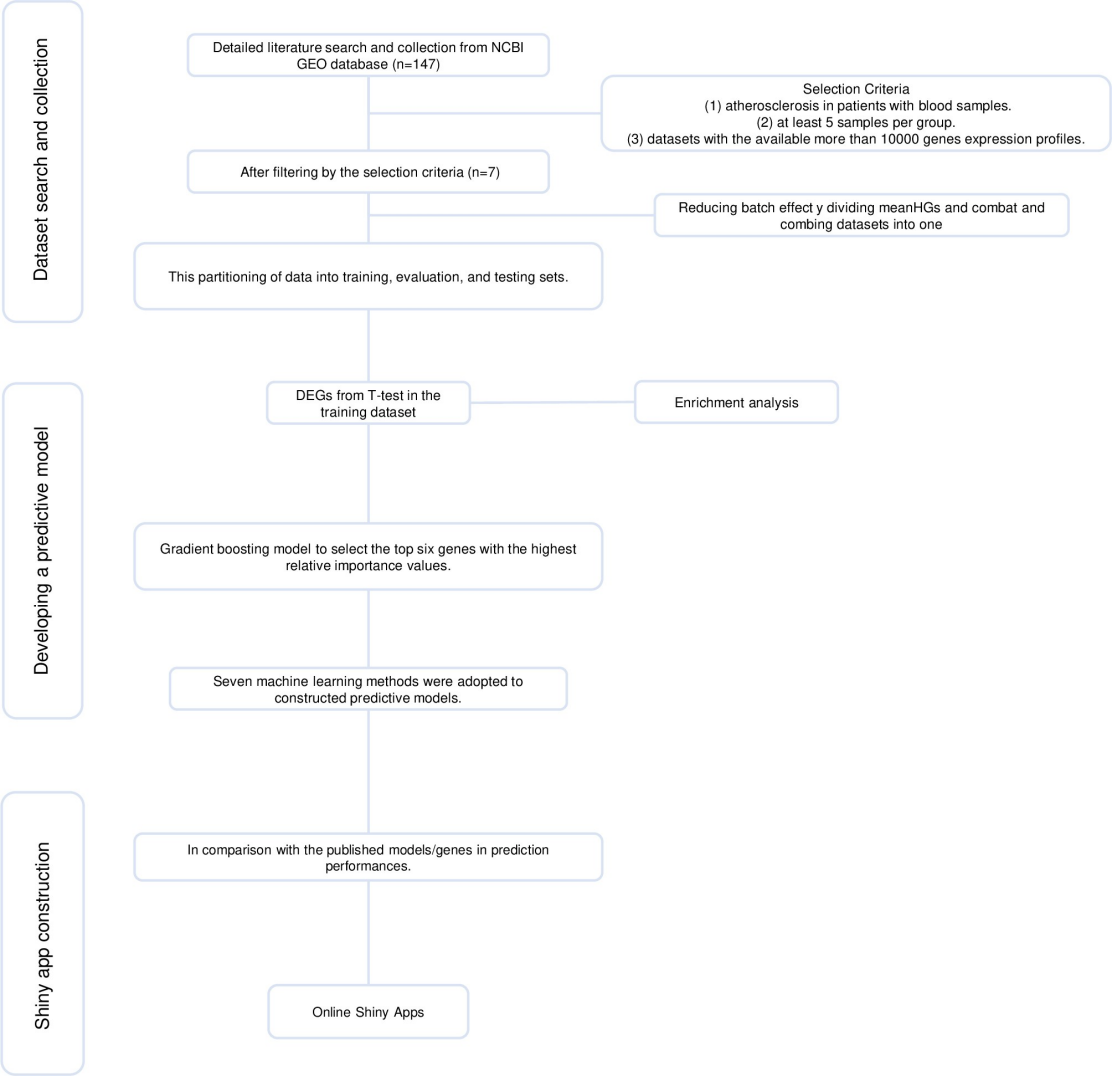
The flowchart of this study.

**Fig 2:**
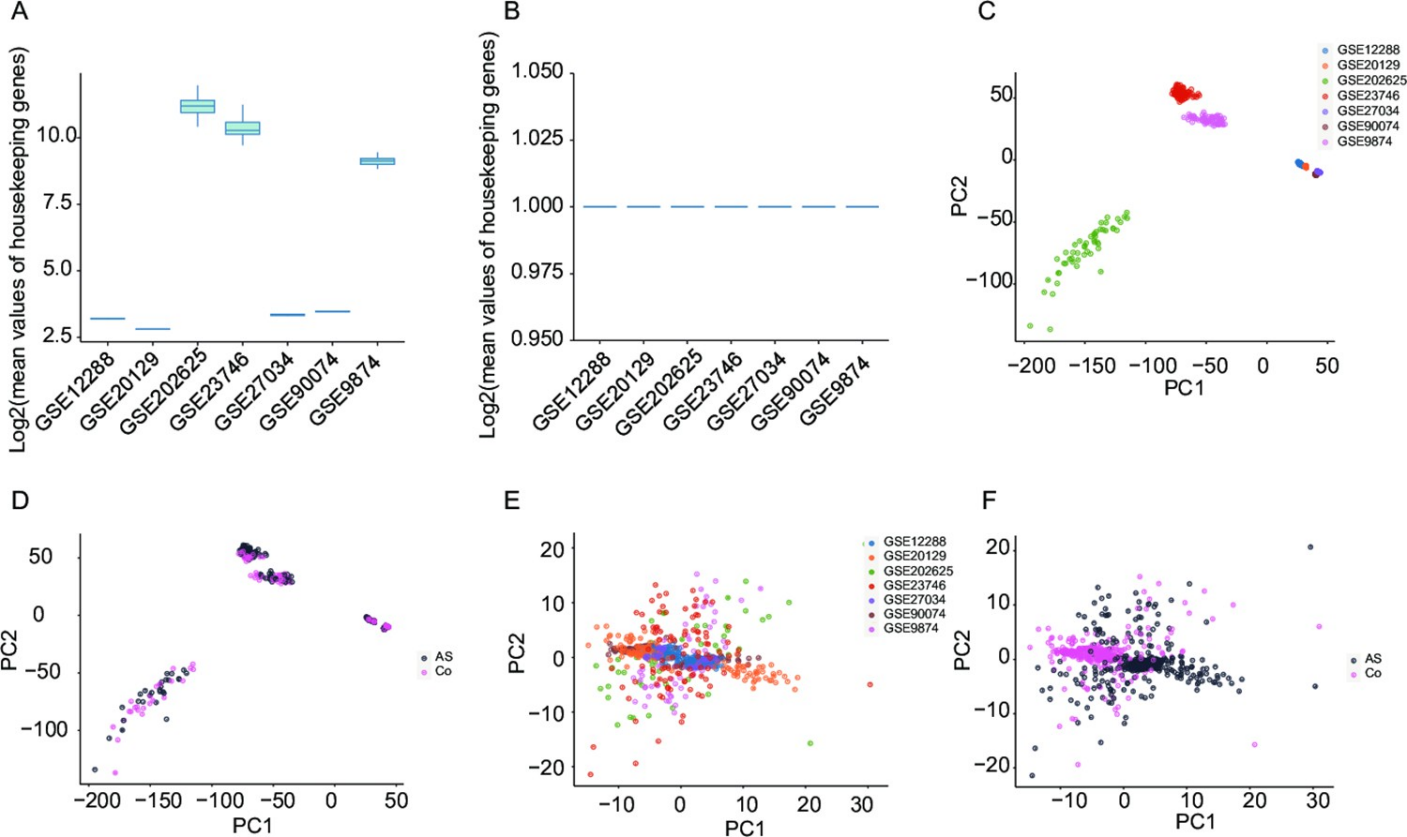
Data pre-processing by standardization (dividing the mean values of housekeeping genes) and batch effect correction. (a) Before the standardization, the mean values of housekeeping genes in samples from different datasets were calculated. (b) After the standardization, the mean values of housekeeping genes in samples from different datasets were calculated. (c) PCA plot showing the distribution of samples from seven studies. Despite standardized bioinformatic processing, samples differed significantly across studies. (d) PCA plot showing the distribution of health controls (Co) and AS samples before batch correction. (e) After applying ComBat, the apparent batch effect was removed. (f) PCA plot showing the distribution of healthy controls (Co) and AS samples from seven studies after the batch correction.

The flowchart of our study is shown in **Fig 1**. We reviewed the available mRNA expression data from the NCBI GEO database. A total of seven GEO datasets (GSE9874, GSE12288, GSE20129, GSE23746, GSE27034, GSE90074, and GSE202625) were filtered based on the requirements provided in the Section “**2.1.Collection of mRNA Expression Data**”. From these seven datasets, there are 728 samples, comprising 403 AS samples and 325 samples from healthy controls. The normalized expression data was directly downloaded for GSE9874, GSE12288, GSE20129, GSE27034, and GSE90074. For GSE23746 and GSE202625, non-normalized expression data were available in the NCBI GEO database. Boxplot showed the overall expression values of samples post-standardization by splitting the meanHGs (**S1A–S1G Fig**). Before dividing the meanHGs, the values of meanHGs in each sample across datasets differed (**Fig 2A**). After dividing the meanHGs, the values of meanHGs in all samples in each dataset were normalized into 1 (**Fig 2B**). Before the application of correction, the batch effect among datasets was significant (**Fig 2C**) since samples of different datasets were clustered differently. The samples from AS and control groups were clustered together (**Fig 2D**), making it challenging to find biomarkers to distinguish AS and control samples. After the application of correction by dividing the meanHGs and using the ComBat function, the batch effects in the datasets were removed (**Fig 2E**). Samples from different datasets were clustered together, and samples from AS and control groups were clustered differently (**Fig 2F**), making it possible to find biomarkers to distinguish AS and control samples. Similarly, the T-SNE result also showed a significant batch effect (**S2A and S2B Fig**), which was removed by dividing the meanHGs and using the ComBat function (**S2C and S2D Fig**).

### 3.2. Data division and identification of DEGs

Among seven datasets, samples from GSE202625 and GSE12288 were selected as the independent testing dataset. Among the samples from the remaining five datasets, 50% were randomly chosen as the training dataset, and the remaining 50% were selected as the evaluation dataset. Finally, there are 227 samples (133 AS and 94 control samples) in the training dataset, 227 samples (133 AS and 94 control samples) in the evaluation dataset, and 274 samples (137 AS and 137 control samples) in the testing dataset. After that, 331 DEGs were distinguished from the training dataset. Among these, 175 upregulated genes and 156 downregulated genes in AS samples were screened out.

### 3.3. Enrichment analyses

DEGs were enriched by WikiPathways, biological processes (BP), and Hallmark. The WikiPathways analysis demonstrated that the up-regulated genes significantly increased in the regulation of toll-like receptor signaling pathway and the chemokine signaling pathway (**S1 Table**). In addition, Hallmark analysis showed that the up-regulated genes in DEGs were enriched in TNF-alpha signaling via NF-kB and interferon gamma response. BP analysis showed that the up-regulated genes were mainly enriched in the cytokine-mediated signaling pathway (GO:0019221) and neutrophil degranulation (GO:0043312). The down-regulated genes had no significantly enhanced WikiPathways, BP, or Hallmark terms.

### 3.4. Classification powers of novel candidate biomarkers

**Fig 3:**
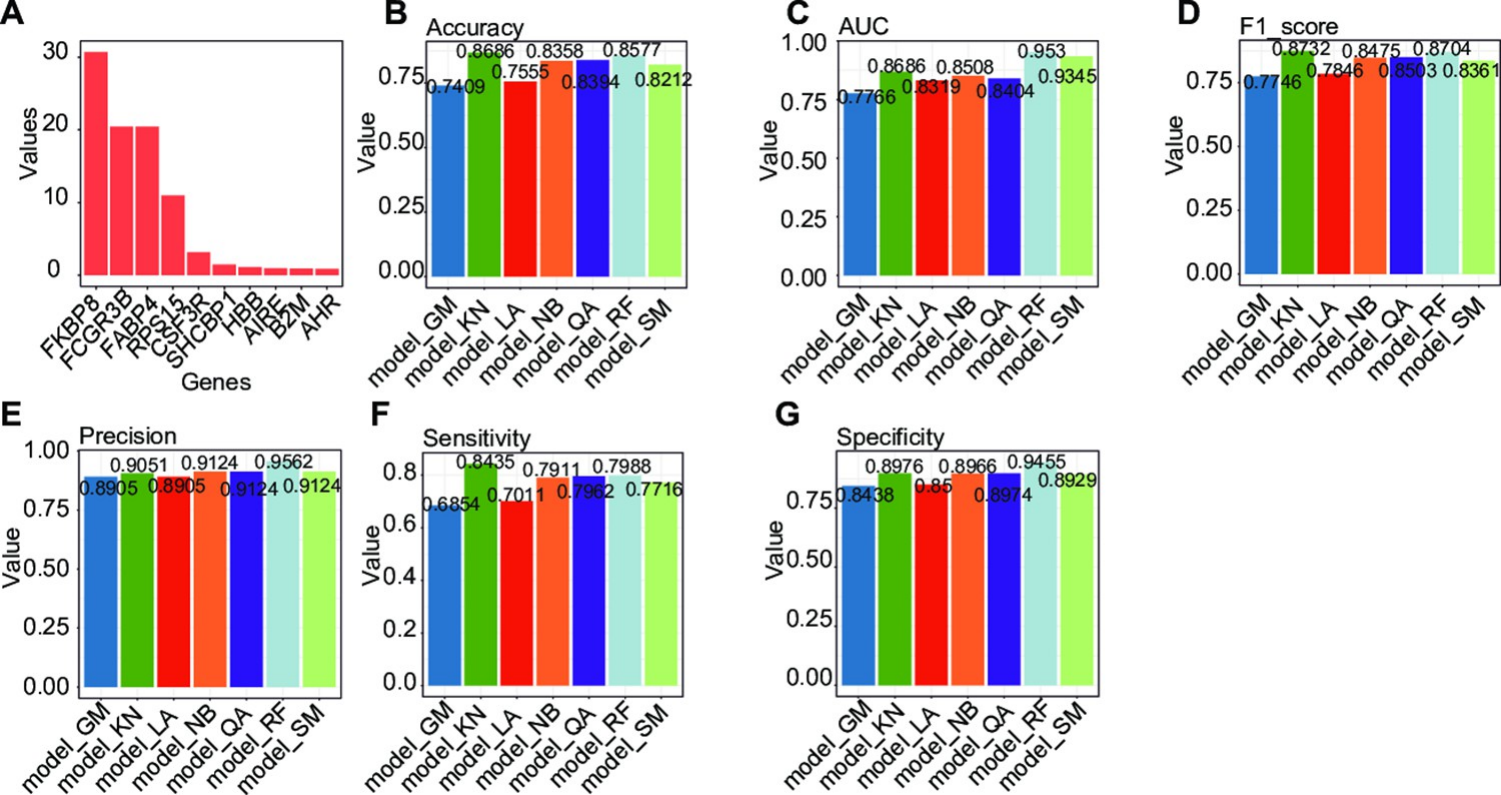
Important genes selection and machine learning validation in the testing dataset. (a) The top 10 genes with the highest relative importance values. Accuracy values (b), AUC (c), F1 score (d), Precision value (e), Sensitivity (f), and Specificity (g) for the best performing model in the testing dataset.

A gradient boosting model analysis was performed based on the gene expression of DEGs to obtain the diagnostic marker genes. We used the gradient boosting model algorithm to screen the diagnostic markers from DEGs. **Fig 3A** illustrates the relative importance values of the top 10 genes. Based on their highest relative importance, the selected genes for model construction are *FKBP8*, *FCGR3B*, *FABP4*, *RPS15*, *CSF3R*, and *SHCBP1*. The effectiveness of potential biomarkers is gauged by their ability to differentiate between the diseased group and controls. Therefore, various classification algorithms, a subset of machine learning techniques, were employed to evaluate these novel biomarkers’ potential, which had been initially identified through statistical tests. The optimal tuning parameters for the seven machine learning models were determined using 5-fold cross-validation on the training dataset. They were subsequently tested on both evaluation and testing datasets to ensure the models’ robustness and generalizability.

For the evaluation dataset, the accuracy values of ’model_GM’, ’model_KN’, ’model_LA’, ’model_NB’, ’model_QA’, ’model_RF’, and ’model_SM’ were 0.9339, 0.9736, 0.9339, 0.9383, 0.9471, 0.9912, and 0.9648 (**S3A Fig**). The AUC values of ’model_GM’, ’model_KN’, ’model_LA’, ’model_NB’, ’model_QA’, ’model_RF’, and ’model_SM’ were 0.9847, 0.9712, 0.9833, 0.9857, 0.9870, 0.9998, and 0.9966 (**S3B Fig**). The F1_score values were 0.9430, 0.9776, 0.9438, 0.9478, 0.9556, 0.9925, and 0.9699 (**S3C Fig**). The Precision values were 0.9323, 0.9850, 0.9474, 0.9549, 0.9699, 0.9925, and 0.9699 (**S3D Fig**). The Sensitivity values were 0.9538, 0.9704, 0.9403, 0.9407, 0.9416, 0.9925, and 0.9699 (**S3E Fig**). The Specificity values were 0.9072, 0.9783, 0.9247, 0.9348, 0.9556, 0.9894, and 0.9574 (**S3F Fig**).

For the testing dataset, the accuracy values of ’model_GM’, ’model_KN’, ’model_LA’, ’model_NB’, ’model_QA’, ’model_RF’, and ’model_SM’ were 0.7409, 0.8686, 0.7555, 0.8358, 0.8394, 0.8577, and 0.8212 (**Fig 3B**). The AUC values of ’model_GM’, ’model_KN’, ’model_LA’, ’model_NB’, ’model_QA’, ’model_RF’, and ’model_SM’ were 0.7766, 0.8686, 0.8319, 0.8508, 0.8404, 0.9530, and 0.9345 (**Fig 3C**). The F1_score values were 0.7746, 0.8732, 0.7846, 0.8475, 0.8503, 0.8704, and 0.8361 (**Fig 3D**). The Precision values were 0.8905, 0.9051, 0.8905, 0.9124, 0.9124, 0.9562, and 0.9124 (**Fig 3E**). The Sensitivity values were 0.6854, 0.8435, 0.7011, 0.7911, 0.7962, 0.7988, and 0.7716 (**Fig 3F**). The Specificity values were 0.8438, 0.8976, 0.8500, 0.8966, 0.8974, 0.9455, and 0.8929 (**Fig 3G**). The classification results showed that the proposed novel biomarkers we have provided here can efficiently discriminate the diseased samples from the controls. Random Forest (RF), which achieved better accuracy and AUC values than other models was selected for further analysis. Upon analysis of our results, we observed a noteworthy disparity between the AUC and accuracy values in the evaluation and testing datasets. Specifically, the metrics were markedly higher in the evaluation dataset compared to the testing dataset. This discrepancy can primarily be attributed to the evaluation dataset being sourced from the same cohort as the training dataset. In contrast, the testing dataset originated from an independent source. These findings underscore the critical importance of utilizing an independent dataset for testing.

### 3.5. Comparison of our models with other studies

**Fig 4:**
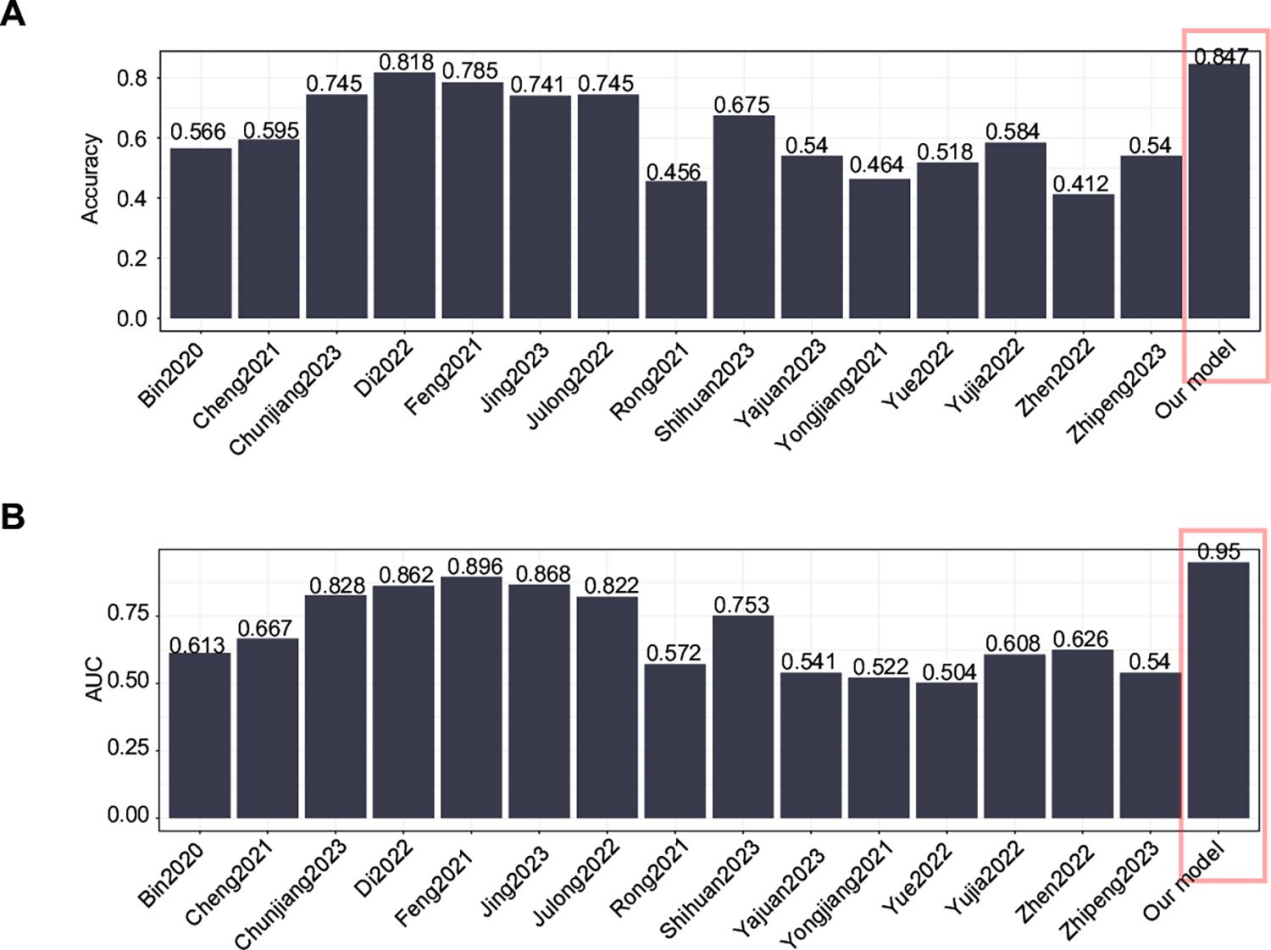
Comparison of our models and other studies in the testing dataset. (a) Accuracy values of models (b) AUC values of models.

**Fig 4A and 4B** shows the accuracy and AUC values difference between our model and other studies. Similar to our approach, these studies leverage publicly available gene identification and model construction datasets. Before the comparison, the genes used in these articles were obtained. In our comparative analysis, we developed models incorporating various genes identified in previous studies into our training dataset, subsequently evaluating their performance on our testing dataset. During the model training phase, we employed 5-fold cross-validation to optimize the parameters (random search) for the Random Forest (RF) algorithm. The models, including ours, were newly built. Our model achieved an accuracy of 0.847, higher than the others, which were 0.818 (Di 2022) and 0.785 (Feng 2021). Similarly, our model achieved the highest AUC value of 0.950 in the testing dataset, followed by 0.896 (Feng 2021) and 0.868 (Jing 2023). However, new datasets are needed to compare these models fairly.

### 3.6. Development of the Shiny app

**Fig 5:**
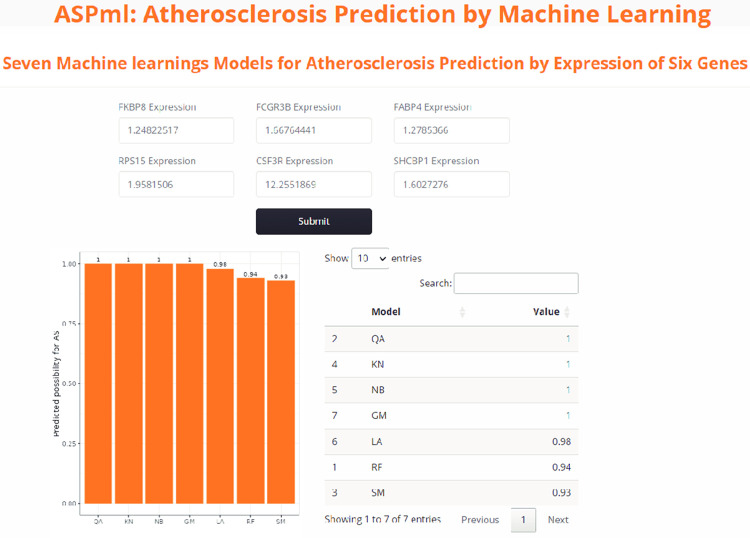
An example of using the Shiny app for atherosclerosis diagnosis. Users are required to enter the expression values of six specific genes and then click the ’Submit’ button. The possibility value of AS will be presented in a table format. The possibility values range from 0 to 1, with a higher value suggesting an increased likelihood of AS.

Shiny apps are built using two main components: a user interface (UI) and a server. The UI defines the layout and appearance of the app, while the server contains the logic and functionality of the app. We uploaded the seven machine learning models to the server. Users could easily input the expression values of six genes and then receive the prediction results from the models (**Fig 5**). The output results contain the table and plot for virilization. The address of the Shiny app is https://zzubioinfo.shinyapps.io/ASPml/. The required input for this Shiny app is the expression values of six genes.

## 4. Discussion

Atherosclerosis is a disease that primarily presents without noticeable symptoms and is difficult to detect accurately. Moreover, atherosclerosis is a significant contributor to cardiovascular diseases, including ischemic heart disease (IHD) and ischemic stroke. It is noted that these cardiovascular diseases rank among the leading causes of death and disability globally [38]. Therefore, detecting atherosclerosis at its earliest stage is crucial, as it facilitates the timely initiation of primary preventive treatments [39]. Hence, discovering atherosclerosis-associated biomarkers is vital for effectively managing cardiovascular disorders [30]. Despite numerous efforts, an accurate and convenient tool for diagnosing atherosclerosis is still unavailable. Therefore, there is an urgent need to identify potential diagnostic markers and construct a machine learning model. We documented 331 DEGs between AS and normal samples in the present work. These DEGs were mainly enriched in immune related pathways, such as neutrophil degranulation, neutrophil activation involved in immune response, and neutrophil mediated immunity. The gradient boosting model obtained six hub genes. Finally, we selected seven machine learning models based on six hub genes, which could accurately discriminate individuals with AS from those with normal samples.

The removal of the batch effect is a major problem when multi-center studies are integrated. Many methods were provided for addressing this problem. These non-biological variables among datasets, such as platforms and techniques, can hinder biomarker identification between AS and normal samples. Also, the machine learning model prediction ability can be seriously reduced by batch effect. Some bioinformatics strategies such as comBat function have been developed for microarray data batch correction [12]. The combat function proposes parametric and non-parametric empirical bayes frameworks for adjusting data for batch effects that are robust to outliers in small sample sizes and perform comparably to existing methods for large samples [13]. The normalization method provided by our study, which divides the housekeeping genes, should be used before applying the comBat function. This strategy may have indirectly contributed to the improved accuracy of our machine learning models in the testing dataset. This is one of the potential disadvantages of using comBat for batch effect reduction. Besides, the performance of ComBat can be sensitive to its parameter settings, such as the prior distributions used for the empirical Bayes estimation. Incorrect parameterization can lead to inadequate correction or artificial introduction of data artifacts. However, it’s imperative to acknowledge that machine learning models can be rendered ineffective in the presence of significant batch effects within the testing dataset. Ultimately, we struck a balance and prioritized the reduction of batch effects to ensure the reliability and generalizability of our results. While ComBat is powerful, its application indeed warrants a discussion of potential limitations or biases, especially when integrating datasets from diverse platforms.

This study obtained six core genes (*FKBP8*, *FCGR3B*, *FABP4*, *RPS15*, *CSF3R*, and *SHCBP1*) that may function importantly during AS occurrence. *FKBP8* (FK506 binding protein 38) is an essential anti‐apoptotic protein because of its interactions with Bcl‐2 [40]. A previous study has found that *FKBP8* is significantly correlated with the extent of coronary atherosclerosis [32]. *FABP4*, referred to as adipocyte fatty-acid-binding protein, is a cytosolic fatty acid chaperone predominantly and expressed in adipocytes and macrophages. Elevated levels of circulating *FABP4* are biomarkers associated with inflammation, hypertension, and cardiovascular incidents [41]. The mechanism of *FABP4* in AS is its direct influence on lipid metabolism coupled with its capacity to mediate inflammatory reactions [42]. *CSF3R* (Colony Stimulating Factor 3 Receptor) acts as a receptor for colony stimulating factor 3, a cytokine responsible for the regulation of production and function of granulocytes. This protein is one of the family of cytokine receptors and plays a role in cell surface adhesion or recognition. A study has reported the correlation of *CSF3R* and Atherosclerosis [43]. The roles of *FCGR3B*, *RPS15*, and *SHCBP1* in AS are poorly understood.

Our study has several strengths. Firstly, we obtained a substantial dataset that contains samples from multiple centers. When used for training, such a comprehensive dataset rendered our model with commendable generalizability, as evidenced by its performance on the validation and testing dataset. Secondly, we conducted experiments comparing our model with those previously released. The results of these comparisons support our claim that our model outperforms other models. Finally, we’ve ensured that our model is both obtainable and open to researchers. Our study also presents several limitations. Firstly, although the machine learning models showed excellent performance, we did not explore their capability by adding new features, such as laboratory test results and clinical variables. The integration of demographic details like age and gender has not been accomplished yet, but such additions might elevate the model’s accuracy. We are currently strategizing for future endeavors to enhance our model’s performance. Secondly, the mechanisms by which these six genes regulate atherosclerosis (AS) require further investigation.

## 5.Conclusion

In summary, using a new integration method, we increased the sample size by batch effect reduction and identified the hub genes between AS and normal samples. These genes were mainly implicated in many immune pathways. The identified six hub genes (*FKBP8*, *FCGR3B*, *FABP4*, *RPS15*, *CSF3R*, *and SHCBP1*) may function importantly in AS occurrence, which may be used as biomarkers for early diagnosis of AS. The online shiny app, constructed using our machine learning models, could contribute to atherosclerosis diagnosis and risk assessment at a very early stage.

## Supporting information

S1 Fig Boxplots of the expression profiles after consolidation and standardization (dividing the meanHGs values). The x-axis represents the sample group, and the y-axis represents gene expression values. The black line in the boxplot represents the median value of gene expression. (a) GSE9874, (b) GSE12288, (c) GSE20129, (d) GSE23746, (e) GSE27034, (f) GSE90074, (g) GSE202625.
(TIF)

S2 Fig T-distributed stochastic neighbor embedding (T-SNE) plot of the gene expression data before (a, b) and after batch correction (c, d). Each point is a sample that is colored according to its batch or group of origin.
(TIF)

S3 Fig Machine learning validation in the evaluation dataset. Accuracy values (a), AUC (b), F1 score (c), Precision value (d), Sensitivity (e), and Specificity (f) for the best performing model in the evaluation dataset.
(TIF)

S1 Table Functional enrichment analysis for the up-regulated DEGs. (CSV)
