# Supplementary figures and images for: Online application for the diagnosis of atherosclerosis by six genes
Source: PLoS One. 2024 Apr 10;19(4):e0301912. doi: 10.1371/journal.pone.0301912 (PMC11006159; doi:10.1371/journal.pone.0301912)

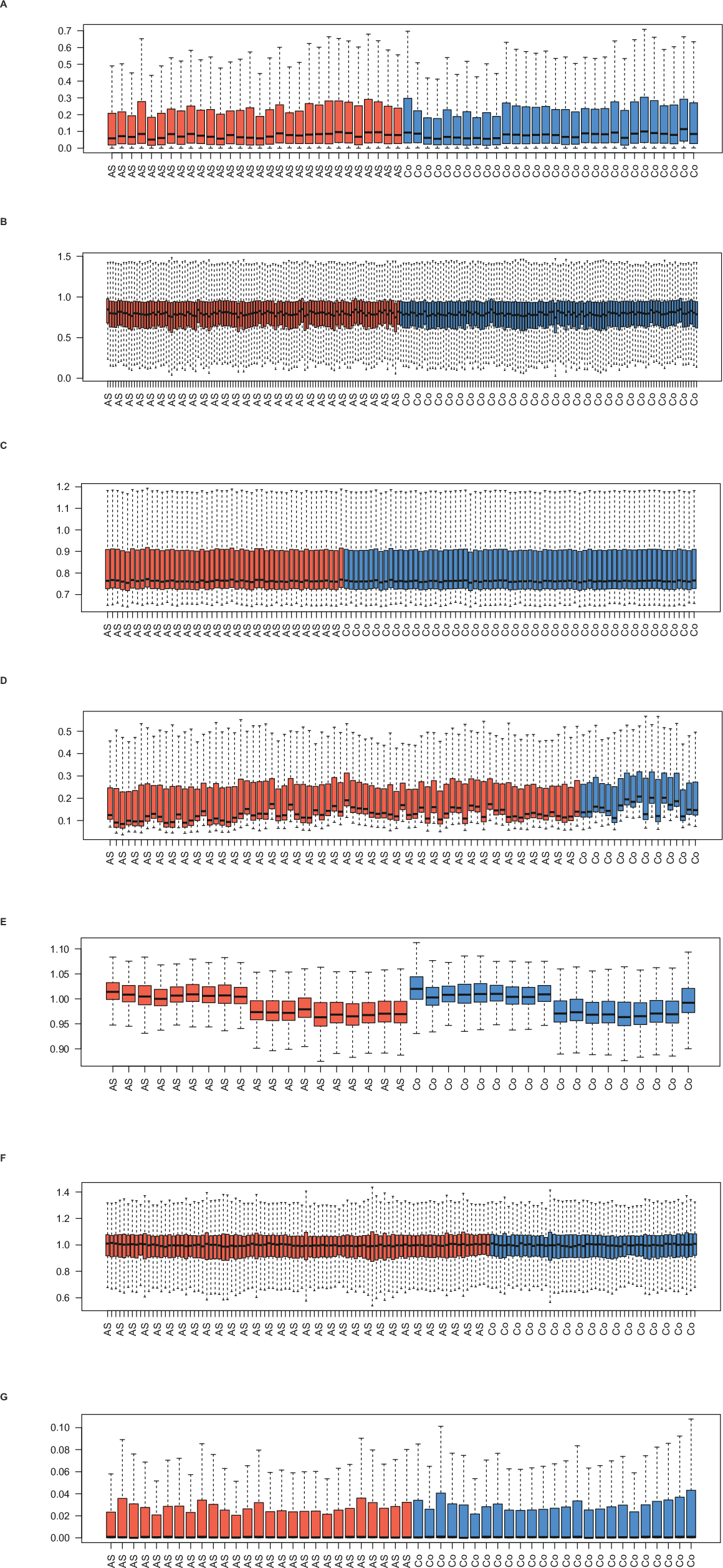

Supplement: S1 Fig — The x-axis represents the sample group, and the y-axis represents gene expression values. The black line in the boxplot represents the median value of gene expression. (a) GSE9874, (b) GSE12288, (c) GSE20129, (d) GSE23746, (e) GSE27034, (f) GSE90074, (g) GSE202625. (TIF) [file pone.0301912.s001.tif]

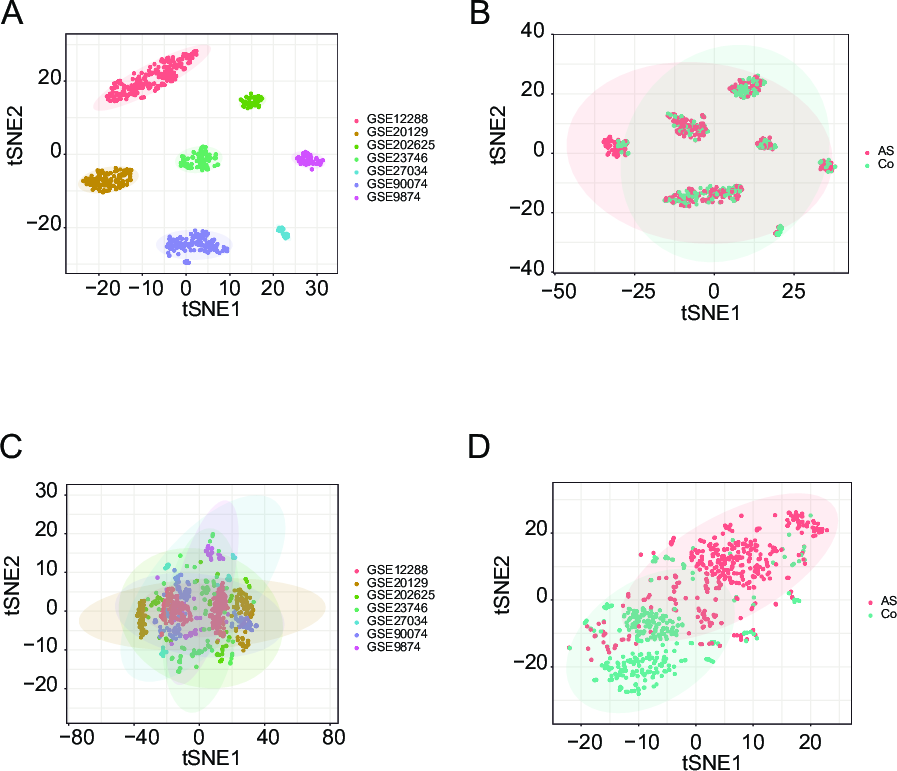

Supplement: S2 Fig — T-distributed stochastic neighbor embedding (T-SNE) plot of the gene expression data before (a, b) and after batch correction (c, d). Each point is a sample that is colored according to its batch or group of origin. (TIF) [file pone.0301912.s002.tif]

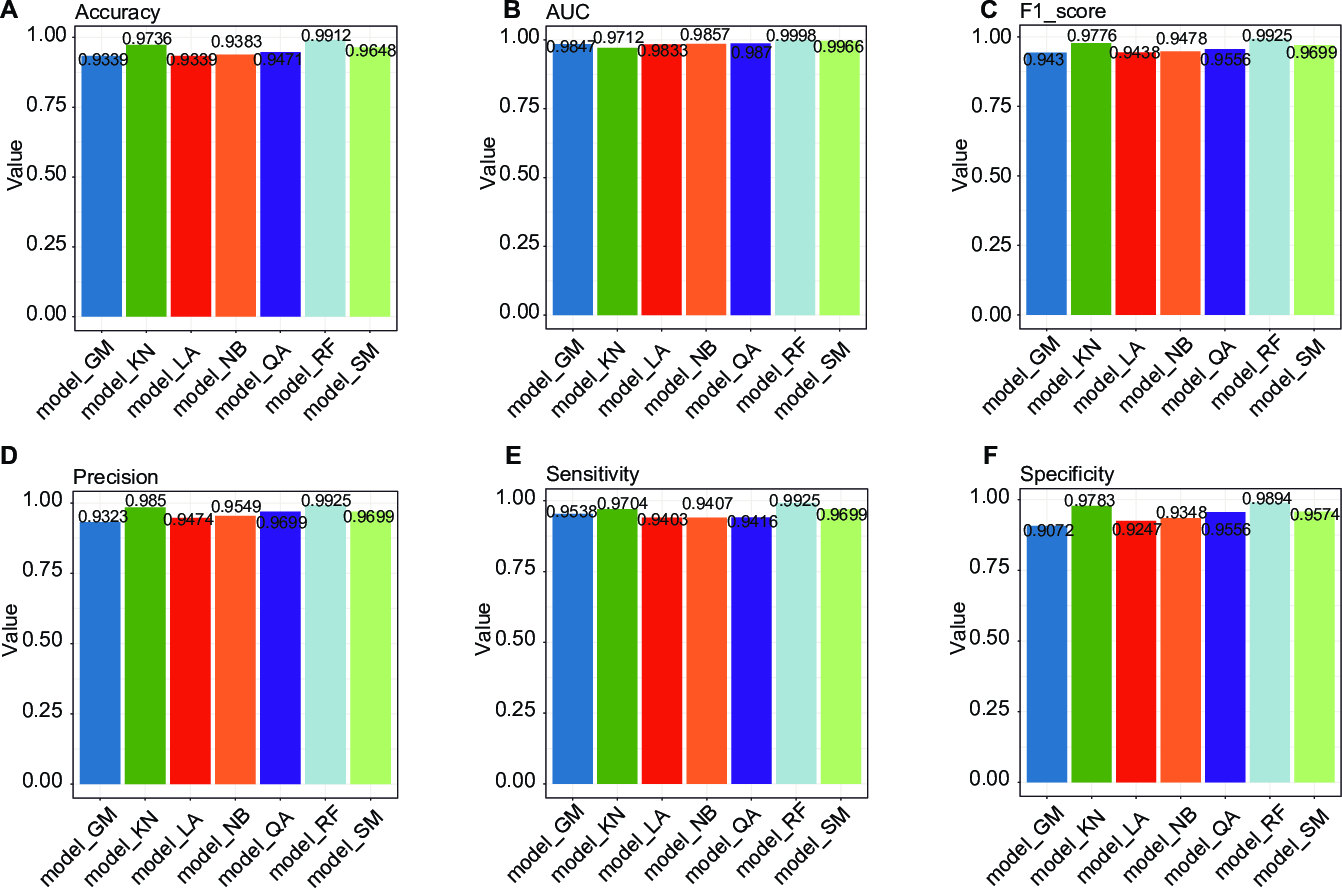

Supplement: S3 Fig — Accuracy values (a), AUC (b), F1 score (c), Precision value (d), Sensitivity (e), and Specificity (f) for the best performing model in the evaluation dataset. (TIF) [file pone.0301912.s003.tif]
